# Microplastics and male reproductive system: A comprehensive review based on cellular and molecular effects

**DOI:** 10.1016/j.toxrep.2026.102226

**Published:** 2026-02-19

**Authors:** Mohammad Karimian, Sahar Yaqubi

**Affiliations:** Department of Molecular and Cell Biology, Faculty of Basic Sciences, University of Mazandaran, Babolsar, Iran

**Keywords:** Microplastics, Human health, Infertility, Spermatogenesis, Cell signaling

## Abstract

Microplastics (MPs) have emerged as an important environmental challenge and can threaten human health. These particles can enter the human body through food consumption, breathing and even skin absorption and cause disruption in the functioning of various organs. In addition, MPs also have an effect on reproduction and can have a negative effect on various stages of reproduction, including gametogenesis and fertilization to embryo formation, and as a result, aggravate infertility problems. This research has investigated the effects of MPs on the male reproductive system, focusing on cellular and molecular processes and approaches to deal with this issue. This review conducted a comprehensive literature search across multiple scientific databases. Searches were performed in Google Scholar, Scopus, PubMed, and Web of Science to identify relevant publications. The search terms used included a combination of MPs, male infertility, sperm, toxicity, and related keywords. The final set of selected articles provides a comprehensive overview of the effects of MPs on cellular and molecular processes in the male reproductive system. In men, MPs can affect the structure and function of the testis and induce the aging process and the inflammatory signaling pathway, oxidative stress, and testicular malignancy. Also, these particles affect the process of spermatogenesis and disrupt sperm parameters. MPs activate different cell signaling pathways and have effects including reducing ATP production, reducing sperm DNA integrity, impairing sperm function and reducing sperm survival. On the other hand, MPs may have destructive effects on the production and balance of male hormones through interaction with the endocrine system. It is evident that proactive measures need to be implemented to address this issue and enhance reproductive health parameters.

## Highlights

• Microplastics influence reproductive health by affecting vital processes such as gametogenesis.
• Specifically, in men, microplastics can negatively affect testicular structure and function.
• Microplastics accelerate aging, trigger inflammation, and provoke oxidative stress in the testes.

## Graphical Abstract

**Figure:**
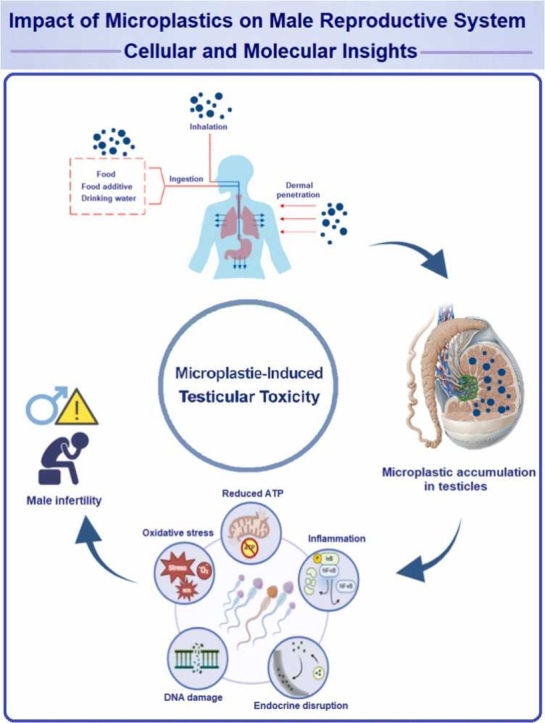

## 1 Introduction

Plastics are not biodegradable and even after hundreds or thousands of years, they do not completely decompose into safe and neutral compounds [1]. Plastic waste can be seen in nature in different shapes and sizes, and it is impossible to remove all of them from the environment. In the meantime, the biggest problem in nature is related to a major group of plastic waste that has a size less than 5 µm and is known by the general name of microplastics (MPs) [2]. MPs are classified into two main categories based on their origin. Some are intentionally manufactured at very small sizes during industrial processes and are referred to as primary MPs. These particles are widely used in everyday personal care and cosmetic products. Owing to their minute size, they can pass through wastewater treatment systems and are consequently released directly into natural environments. In contrast, secondary MPs are formed through the gradual degradation and fragmentation of larger plastic materials under the influence of various environmental factors, after which they become widely distributed across diverse ecosystems [3], [4], [5]. MPs, whether alone or combined with other toxic pollutants, interfere with cellular energy production and metabolism, promote oxidative stress, and induce a range of harmful effects, including cancer and endocrine disruption [6]. Moreover, their impact extends beyond the endocrine system, affecting the digestive, respiratory, immune, cardiovascular, nervous, and reproductive systems [7], [8].

The testis appears to be the most sensitive organ to many environmental toxicants due to its unique functional processes [9]. Spermatogenesis is a highly complex process that occurs within the seminiferous tubules of the testes; this process requires a highly specialized microenvironment to support the growth and maturation of sperm cells, which is provided by somatic cells such as Sertoli, Leydig, and peritubular myoid cells [10]. Any disruption of this process can have significant consequences for sperm quality and male fertility potential [11]. In addition, the blood-testis barrier (BTB), formed by adjacent Sertoli cells near the basement membrane, acts as a “gatekeeper” to prevent harmful substances from reaching developing germ cells, particularly post-meiotic spermatids [12]. Nevertheless, increasing evidence suggests that many emerging pollutants, including MPs, interact with this complex barrier, traverse it, and compromise its integrity [13]. MPs not only disrupt testicular structure and function but also induce oxidative stress, apoptosis, cellular senescence, and disturbances in cellular energy homeostasis by creating a pro-inflammatory environment within the testicular microenvironment [14].

Since MPs can enter cells, they exert their effects by influencing cellular and molecular pathways. For example, by affecting the mTOR signaling pathway, they may lead to disruption of BTB integrity [15]. Alternatively, through activation of the NF-κB signaling pathway and the secretion of pro-inflammatory cytokines, they can induce aging and inflammatory processes in the testes [16]. In addition, free radicals generated by MPs can activate certain cellular signaling cascades associated with oxidative stress, which may cause damage to the male reproductive system [17]. Some evidence even suggests that MPs may induce malignancies in the testes [18]. Despite the growing body of experimental evidence in recent years, a comprehensive understanding of the cellular and molecular mechanisms involved in microplastic-induced reproductive toxicity, particularly in relation to male infertility, remains lacking. This knowledge gap highlights the need for focused review studies on the effects of MPs on testicular structure and function as well as the process of spermatogenesis. The aim of this study is to describe the effects of MPs on the male reproductive system with an emphasis on cellular and molecular processes.

## 2 Methods

This review involved a comprehensive literature search across multiple scientific databases. Searches were conducted in PubMed, Google Scholar, and Web of Science up to December 2025 to identify relevant publications. The search strategy in each database included combinations of the keywords “microplastics”, “MPs”, “male infertility”, “sperm”, “toxicity”, and other related terms to ensure a thorough coverage of the topic. The final set of selected articles provides a detailed overview of the current state of research on the effects of MPs on cellular and molecular processes in the male reproductive system.

This article is divided into two main sections. The first part explores the growing challenge posed by MPs in environmental pollution and their impact on human health. It examines the harmful effects of MPs on reproductive health, with a focus on the testis, including their influence on the BTB, the induction of aging processes, inflammatory pathways, and oxidative stress. Furthermore, the association between MPs and testicular malignancies is discussed.

The second part delves into the effects of MPs on the process of spermatogenesis. It highlights their impact on sperm parameters, genome integrity, and the cells involved in spermatogenesis. Additionally, it reviews the influence of MPs on sex hormones and their role in endocrine disorders. The article concludes by discussing strategies to address the challenges posed by MPs in human health and environmental contexts.

## 3 Impact of microplastics on the environment and health

MPs are recognized as persistent and widespread pollutants in the environment, present across all ecosystems from deep oceans to soil and air sources. These tiny particles originate from primary sources such as personal care and cosmetic products, as well as from the degradation of larger plastics in the environment. Due to their chemical resistance, MPs readily accumulate in aquatic and terrestrial environments and remain stable for many years [19]. Their widespread presence in water sources and animal feed facilitates their entry into the food chain, ultimately increasing the risk of human exposure [20]. Studies have shown that MPs can cause biochemical disruptions, oxidative stress, and inflammation in aquatic species, and they also act as carriers of pathogenic agents and toxic pollutants, posing potential serious health risks to humans [21], [22]. Therefore, reducing emissions and managing microplastic pollution sources is essential for protecting environmental and human health.

### 3.1 Types of microplastics and their major sources of pollution

Some MPs are intentionally produced at microscopic sizes during industrial manufacturing processes and are referred to as primary MPs. These particles are commonly used in personal care and cosmetic products such as skin cleansers, toothpaste, creams, and sunscreens. Due to their small size, they can bypass wastewater treatment systems and are directly released into the environment. In contrast, secondary MPs are generated through the gradual fragmentation of larger plastic items under the influence of environmental factors such as ultraviolet radiation, physical abrasion, wave action, and biological activity, after which they become widely dispersed across different ecosystems [4], [5].

Land-based sources are considered the dominant contributors to microplastic contamination, accounting for approximately 80–90 % of MPs entering aquatic environments [23]. Urban plastic waste, packaging materials, personal care products, synthetic textiles, construction materials, and industrial activities represent the major terrestrial sources. The release of synthetic fibers during laundering, abrasion of vehicle tires, sewage sludge application, and residues from waste incineration significantly facilitate the transport of MPs from land into rivers and marine systems [24], [25]. In addition, the widespread consumption of single-use plastic products in recent years has further intensified microplastic pollution [26].

Alongside terrestrial inputs, ocean-based sources also contribute substantially to microplastic pollution, accounting for approximately 10–20 % of the total MPs directly released into marine environments [27]. Coastal tourism, commercial fishing, maritime transport, and offshore industries are among the principal marine-related sources. The loss or disposal of plastic fishing gear, including nylon nets and monofilament lines capable of remaining buoyant at various ocean depths, represents a major pathway for microplastic entry into marine ecosystems. Furthermore, waste discharges from ships and offshore industrial operations, particularly petrochemical activities, contribute directly to increasing microplastic loads in marine environments [26].

### 3.2 Microplastics and their environmental impacts

MPs are primarily composed of common polymers such as polyethylene, polypropylene, polyvinyl chloride (PVC), polystyrene, polyurethane, and polyethylene terephthalate (PET), with the majority of MPs in the environment consisting of polyethylene, polypropylene, and polystyrene [28]. Due to their high chemical resistance, semi-crystalline structure, and stability against physical and chemical factors, these polymers degrade very slowly and can persist for long periods in water, soil, and sediments [29], [30]. Polystyrene, because of its thermal and chemical stability, is widely used in packaging and insulation, but these same properties cause it to accumulate long-term in natural ecosystems, increasing environmental pollution load [31], [32], [33], [34].

During plastic production, various organic and inorganic additives are added to polymers to enhance properties such as resistance to light, heat, moisture, oxidation, and biodegradation [35]. These additives include fillers, plasticizers, stabilizers, lubricants, flame retardants, and pigments. Mineral fillers such as silica, talc, clay, aluminum oxide, and glass increase the strength of plastics and enhance their environmental persistence [36]. Many of these compounds are highly resistant to degradation and tend to accumulate in soil and water resources [31].

Most additives are not chemically bonded to the polymer chain and can gradually separate from the plastic structure and enter the environment over time [35]. Organic and inorganic pigments used in plastics, especially those containing heavy metals such as lead, cadmium, cobalt, and mercury, are potential sources of contamination in soil, water, and air [37]. Although additives improve the physical and chemical performance of plastics, their gradual release from MPs plays a significant role in increasing environmental pollution and threatening ecosystem sustainability [31].

### 3.3 Microplastics in food chains and their implications for animal and human health

Given the widespread and increasing contamination of environmental habitats by MPs, these particles are increasingly likely to enter animal bodies. MP uptake can occur either directly from the surrounding environment or indirectly through prey, i.e., via transfer along food chains [38]. MPs readily accumulate in biofilter-feeding organisms such as mollusks, ascidians, and zooplankton, and they are also capable of infiltrating terrestrial food chains [39]. Numerous studies have investigated the effects of MPs on various aquatic species, particularly invertebrates and fish. In aquatic invertebrates, although most studies have not reported a significant increase in mortality, substantial evidence indicates disruptions in feeding activity and reduced fertility, inhibition of larval growth and development, increased oxygen consumption, and elevated production of reactive oxygen species [40]. Increased mortality has been reported only in certain species, including *Perinereis aibuhitensis*, *Palaemonetes pugio*, and *Tigriotopus japonicus*, and under exposure to specific sizes and concentrations of MPs. However, the wide variability in polymer types, particle sizes and concentrations, as well as differences among studied species, limits direct comparison of the results [39]. In fish, the presence of MPs has been reported in a considerable proportion of specimens collected from the Northeast Atlantic Ocean, with particles predominantly detected in the gastrointestinal tract, gills, and in some cases, muscle tissues [41]. Exposure to MPs has been associated with increased lipid peroxidation, induction of oxidative stress, and altered activity of acetylcholinesterase in neural tissues [41]. Review studies and meta-analyses indicate that smaller particles, particularly polystyrene, exhibit higher toxicity and are capable of crossing biological barriers, undergoing maternal transfer, and affecting early larval development [42], [43], [44]. Increasing exposure doses intensify tissue accumulation and the severity of histopathological and biochemical alterations [45], [46]. Overall, existing evidence suggests that microplastic accumulation, especially in the fish intestine, plays a critical role in triggering inflammation, oxidative stress, alterations in gut microbiota, and metabolic disturbances [39], [43].

A review of experimental studies on the biological effects of microplastic particles in terrestrial mammals indicates that scientific evidence in this field has increased substantially in recent years. Numerous studies have reported exposure of laboratory rodents to MPs, reflecting the growing attention of the scientific community to this emerging environmental challenge [39]. Across these studies, considerable heterogeneity exists in terms of animal species and strains, particle characteristics, exposure duration, dose, and routes of administration. Most experiments were conducted on mice, particularly the ICR (CD-1) and C57BL/6 strains, with fewer studies using Balb/c or Swiss mice, while a smaller proportion employed Wistar or Sprague-Dawley rats. Oral exposure, either via drinking water or gastric gavage, was the most commonly used route, although some studies applied dietary administration, inhalation, intratracheal instillation, or intraperitoneal injection. Polystyrene particles were predominantly investigated, typically within a size range of 0.02–500 μm, whereas polyethylene particles were less frequently studied and generally exhibited larger mean diameters. This methodological diversity should be taken into account when interpreting and comparing the biological outcomes reported across studies [39].

The widespread presence of MPs in animal feed, water resources, and environmental matrices represents a major indirect pathway for human exposure, as these particles are capable of accumulating in edible animal tissues and seafood products [47]. Numerous reports have documented the detection of plastic particles in livestock feed, animal-derived products, and even blood samples, raising growing concerns regarding food safety and the potential long-term consequences for human health [47]. In addition to direct dietary intake, MPs may exert indirect effects on human health by acting as carriers of toxic substances and pathogenic agents. Available evidence indicates that these particles can adsorb and transport pathogenic microorganisms, heavy metals, and persistent organic pollutants, thereby increasing the risk of gastrointestinal disorders, systemic toxicity, and potential carcinogenic processes [38]. Moreover, chronic exposure to high levels of MPs has been associated with alterations in gut microbiota composition, increased intestinal permeability, induction of inflammatory responses, and the development of endotoxemia. Collectively, these findings highlight the role of MPs as effective biological mediators linking animal and human health and underscore the urgent need to address their presence within food production and supply systems [38], [48], [49].

## 4 The impact of microplastics on male reproductive health

Available evidence indicates that exposure to environmental pollutants can disrupt reproductive function, leading to structural and functional impairment of the reproductive system and a decline in reproductive capacity across different age groups. One of the most important biological pollutants is MPs, which are widely dispersed in the environment and have adverse effects on the reproductive system and reproductive health [50]. Studies show that if rodents are exposed to MPs of different sizes, they can cross the BTB and enter the testicular tissue [51], [52]. The exposure of male mice to MPs led to the creation of abnormal sperms and also reduced the total number of sperms [51]. Also, in a similar study, it was found that polystyrene can lead to teratospermia and oligospermia in mice [53]. It was also found that the oral exposure of male rats to MPs can cause their accumulation in the testicular tissue, and subsequently, the serum testosterone level decreases and sperm viability decreases [52].

**Fig. 1:**
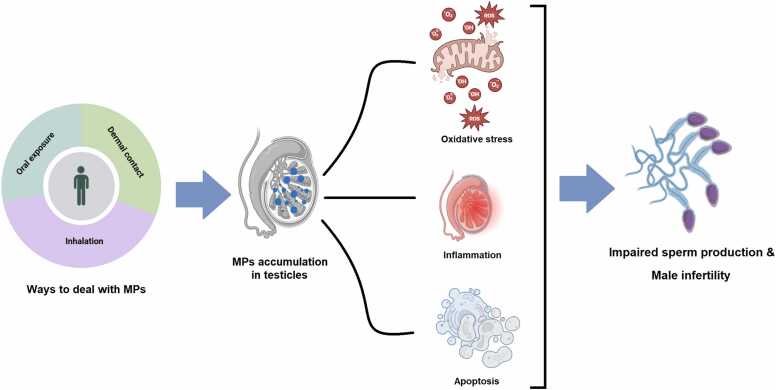
Routes of microplastic entry into the body, their accumulation in the testes, and disruption of spermatogenesis. Exposure to microplastics through various routes, including oral ingestion, inhalation, and dermal contact, may allow these particles to cross physiological barriers and accumulate in the testes. Such accumulation is associated with cellular and molecular alterations, including increased oxidative stress, inflammation, and activation of apoptotic pathways, which may ultimately impair spermatogenesis and contribute to male infertility.

In addition to morphological changes in sperm and reductions in classical fertility indices, new evidence suggests that MPs can affect testicular function at a deeper level by disrupting cellular signaling pathways. It has been shown that after exposure to polystyrene microplastics (PS-MPs), the expression of pro-inflammatory molecules and inflammatory factors significantly increased, while the expression of anti-inflammatory molecules decreased [54]. Also, PS-MPs of 5 µm size have been shown to reduce ATP content, decrease mitochondrial membrane potential, damage the integrity of the mitochondrial genome, and disrupt the homeostatic balance between mitochondrial fission and fusion. Additionally, the PINK1/Parkin-dependent autophagy pathway was activated. Time-series analyses revealed that these particles cause mitochondrial structural damage through induction of cellular oxidative stress, although mitochondrial function is partially maintained after injury. Overall, these findings demonstrate the mitochondrial toxicity of PS-MPs and provide a basis for understanding the mechanisms of sperm damage caused by these particles [55]. PS-MPs also increase oxidative stress in testicular tissues and negatively impact spermatogenesis and fertility in male mice. Long-term exposure to these particles leads to decreased activity of important antioxidant enzymes in testicular tissue. These disruptions ultimately reduce sperm production and fertility (Fig. 1) [56].

## 5 The effect of microplastics on the structure and function of the testis

Continuous exposure to MPs can lead to their accumulation in testicular tissue, disruption of testicular function, and ultimately an increased risk of male infertility [57]. Studies have shown that exposure to PS-MPs can induce pathological alterations in testicular morphology, including abnormal development of seminiferous tubules [58]. In addition, these particles extensively impair testicular structure and function in both young and aged mice, resulting in excessive germ cell loss, seminiferous tubule degeneration, tissue fibrosis, and reduced sperm quality. Notably, young mice exhibit signs of premature testicular aging, whereas older mice display more severe testicular damage, indicating age-dependent susceptibility to testicular injury [59]. Furthermore, a significant negative correlation has been reported between specific polymers, such as PVC and PET, and normalized testicular weight, highlighting the direct impact of these pollutants on testicular structure [60]. Moreover, nanoplastics derived from polylactic acid microplastics (PLA-MPs) have been shown to penetrate the BTB after gastrointestinal degradation and accumulate within the spermatogenic microenvironment. Long-term exposure to these particles is associated with pronounced reproductive toxicity, disruption of sex hormone homeostasis, damage to the BTB, which ultimately exacerbates testicular structural damage [61]. Collectively, these findings demonstrate that MPs, by targeting critical testicular structures such as seminiferous tubules and the BTB, play a significant role in testicular dysfunction and increased risk of male infertility.

### 5.1 Effects of microplastics on testicular blood barrier

**Fig. 2:**
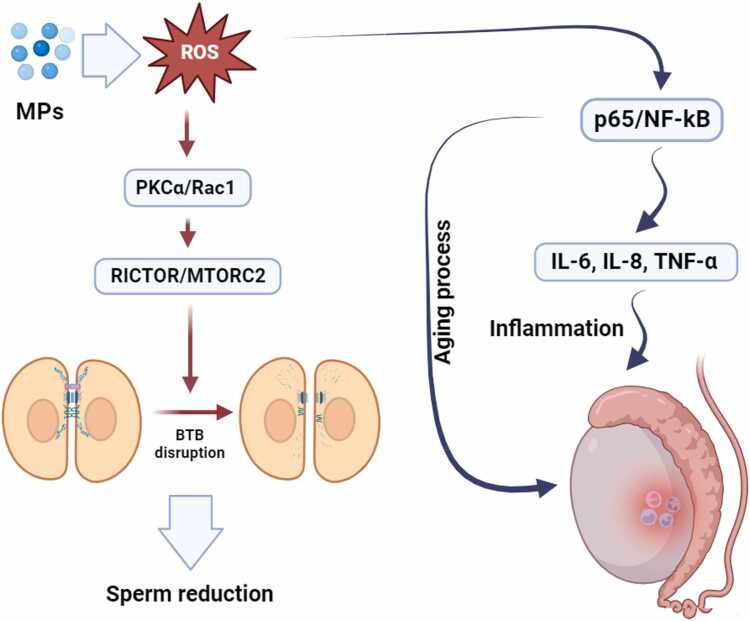
The effect of microplastics on testicular blood barrier disruption, inflammation, and testicular aging. By causing oxidative stress, microplastics can affect PKCα/Rac1 and then RICTOR/MTORC2 pathways, thereby disrupting the cytoskeleton especially actin filaments and cellular junctions, including tight junctions, desmosomes, and gap junctions in the testicular blood barrier and as a result reduce the number of sperms. On the other hand, the oxidative stress caused by microplastics can affect the p65/NF-kB pathway and increase inflammation in the testes through the increase of IL-6, IL-8, and TNF-α. Also, increasing the expression of p65/NF-kB can intensify the aging process in the testes.

Among various environmental MPs, polystyrene-based particles have attracted particular attention due to their pronounced bioavailability and reported reproductive toxicity, especially in the male reproductive system [52], [53]. This compound may exert its toxic effects on the male reproductive system by affecting the BTB and disrupting its integrity. PS-MPs may cause loss of BTB integrity and in turn lead to spermatogenic dysfunction [15]. In the testis, the organization of actin filaments is often controlled by FAK and mTOR [62]. mTOR includes two complexes, mTORC1 and mTORC2, which have two opposing effects on F-actin and control BTB integrity. Rps6/mTORC1 disrupt BTB integrity via Akt/MMP9, and RICTOR/MTORC2 promote BTB integrity via PKCα/Rac1 [63], [64]. ROS suppress the mTOR/Akt signaling pathway [65], which PS-MPs can also produce [66]. Therefore, BTB disruption caused by PS-MPs could be exerted through the mTOR pathway (Fig. 2). Wei et al. (2021) study showed that PS-MPs induced the imbalance of mTORC1 and mTORC2 through ROS explosion, and by changing the expression pattern of actin filament binding proteins, it caused the disintegration of these filaments. These MPs reduced the expression of binding proteins in BTB and finally caused disruption of BTB cohesion and disruption of spermatogenesis process [15].

### 5.2 Induction of aging process and inflammatory signaling pathway

Cellular aging is characterized by irreversible cell cycle arrest, functional decline, and activation of inflammatory signaling pathways such as NF-κB, which are commonly associated with genomic instability, mitochondrial dysfunction, and oxidative stress [67], [68]. It has been found that exposure to environmental pollutants is one of the main causes of accelerating the aging process and related diseases [69]. However, the effect of MPs on testicular aging has not been investigated in detail. In 2023, Wu et al. investigated the effect of PS-MPs on premature aging of testicular tissue [16]. The results of their study showed that premature aging in TM4 Sertoli cells and premature aging of the testis can be induced by PS-MPs. Wu et al. (2023) investigated the involvement of NF-κB signaling pathway in premature testicular aging caused by PS-MPs. It was found that PS-MPs lead to premature aging of testicular tissue and this process is associated with increased expression of p65/NF-kB. On the other hand, when the NF-κB signaling pathway was inhibited, PS-MPs-induced aging in TM4 cells was reduced, which indicates the mediation of the NF-κB signaling pathway in the aging process of the testis. Also, the aging-related secretory phenotype was observed as the downstream effects of NF-κB in senescent cells, which was associated with pro-inflammatory factors such as IL-6, IL-8, and TNF-α (Fig. 2) [16]. Moreover, Polyethylene microplastics (PE-MP) exposure markedly elevated the expression of key pro-inflammatory mediators, including TNF-α, IL-1β, NF-κB, IL-6, and COX-2, indicating a pronounced activation of inflammatory signaling pathways and a sustained inflammatory response [70].

### 5.3 Induction of oxidative stress in the testis by microplastics

**Fig. 3:**
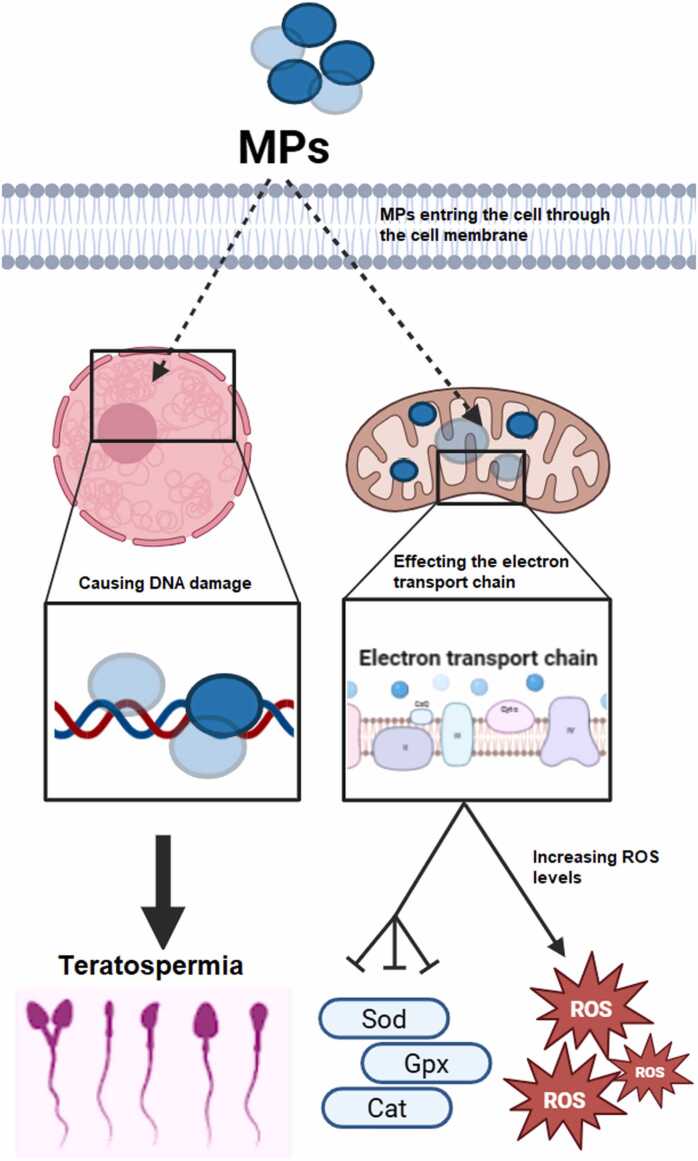
The effect of microplastics on nucleus and mitochondria organelles in the testicular cells. After entering the germ cells, microplastics can enter the nucleus and destroy the integrity of the genome, and disrupt the morphology of the sperms. On the other hand, microplastics, by entering the mitochondria organelle, affect the electron transport chain and cause its disruption, which has negative effects on antioxidant enzymes such as catalase, glutathione reductase, superoxide dismutase, and glutathione peroxidase, in turn, increases oxidative stress.

Oxidative stress arises from an imbalance between prooxidants and antioxidants, leading to the excessive generation of reactive species that can indiscriminately damage nearby biological molecules [71]. By accumulating in the mitochondria and interfering with the electron transport chain, MPs cause damage to the membrane of this organelle and ultimately cause the production of free radicals, which in turn endanger the antioxidant defense reservoir, lipid peroxidation, protein oxidation and DNA damage (Fig. 3) [17], [72]. Also, free radicals caused by MPs can trigger some cellular signaling cascades such as TGF-β pathways, PI3Ks/Akt signaling pathway, Nrf2 pathway, MAPKs signaling pathway including the JNK, p38 kinase, and ERK1/2 signaling cascades, and p53 signaling pathway [17]. Therefore, oxidative stress caused by MPs can lead to the damage of various organs in humans, such as hepatotoxicity, immunotoxicity, renal toxicity, neurotoxicity, cardiotoxicity, pulmonary toxicity, reproductive toxicity, etc. [17].

PE-MP are one of the most common MPs in groundwater and freshwater ecosystems [73]. A large amount of polymers produced around the world is of polyethylene type and more than half of it is used for bottles and synthetic fibers [74]. Humans are exposed to this microplastic in various ways, including skin contact, swallowing, and inhalation [75] and it has adverse effects on several organs such as the testicles [70]. PE-MP can cause testicular damage mainly through oxidative stress. In one study, it was found that treatment of male rats with PE-MP significantly increased the levels of malondialdehyde and reactive oxygen species, as well as the activity of antioxidant enzymes, such as superoxide dismutase, glutathione reductase, catalase, and glutathione peroxidase was decreased [70]. Also, in another study, the effects of another type of microplastic, namely PS-MP, on the production of oxidative stress in rat testes were investigated. Also, the results of this study showed that exposure to this microplastic can decrease the activity of glutathione reductase, glutathione peroxidase, superoxide dismutase, and catalase enzymes (Fig. 3).

Also, some other parameters including the levels of malondialdehyde and reactive oxygen species increase due to PS-MP [76]. The mentioned antioxidant enzymes are considered endogenous, which are known as the first line of cell defense against oxidative stress and ROS reduction, and thus can protect macromolecules such as DNA, lipids, and proteins. Hydrogen peroxide, superoxide anion, hydroxyl radical and nitric oxide are important types of reactive nitrogen and oxygen species [77]. Therefore, when the activity of antioxidant enzymes decreases, the concentration of reactive oxygen species increases and damages the testis in various ways [78]. Polyvinyl chloride is used as a raw material in the manufacture of chemical and plastic industries, including plastic pipes and disposable containers, wires and cables, toys and automotive electronics, etc. [79]. PVC can cause toxicity in tissues such as liver and testis. The results of studies have determined that this substance causes apoptosis in testicular tissue, reducing the activity of antioxidant enzymes and inducing oxidative stress [80], [81]. The study of Sadeghi et al. (2020) showed that polyvinyl chloride can increase the level of malondialdehyde and thus oxidative stress in the rat testis [82]. Of course, some antioxidant compounds can reduce the damage caused by ROS induced by polyethylene and polystyrene [70], [76].

### 5.4 Microplastics and testicular malignancy

Testicular cancer is the most frequently diagnosed malignancy in young men and, despite its relatively low prevalence, represents a significant clinical concern due to its impact on male reproductive function [83], [84]. Previous occupational evidence suggests that certain occupations are potentially associated with an increased risk of testicular cancer. For example, despite the fact that the overall prevalence of cancer is lower among farmers, pesticides can increase the risk of testicular cancer up to three times [85], [86], [87]. In addition, plastic-related industries can increase the risk of testicular cancer. People who are in industries related to the production and manufacture of polyvinyl chloride may have a high risk of testicular cancer [88].

Often, testicular cancer is not considered an occupational disorder. However, a study with a case-control design in order to investigate occupational exposures and testicular cancer showed that exposure to PVC can increase the risk of testicular cancer, especially seminoma, by 6 times. The authors of the paper stated that if cases with self-reported orchitis or cryptorchidism were excluded, a greater increase in risk was observed, and 6 of 7 subjects had seminoma. They also reported that some other plastics had no effect on testicular cancer risk. It was not clear what the reason for the increased incidence of seminoma compared to embryonal malignancy, but considering that seminoma occurs at a later age than embryonal, it may increase the possibility of an occupational correlation. Although seminoma malignancy is generally not considered an occupational disease [18]. Another study looked at the incidence of cancer in workers exposed to vinyl chloride. The results of this cohort study determined that although PVC increases the risk of some malignancies, it does not increase the risk of testicular cancer [89]. Another study found that exposure to plastics, including PVC, could not be associated with an increased risk of testicular cancer. They stated that not necessarily all workers in the plastics industry are actually exposed to plastic compounds and that some incorrect groupings may have occurred in their research, which could have biased their results. Therefore, only those workers who have the most contact with plastic materials should be evaluated [90]. Another case-control study was designed in this field and its results showed that exposure to PVC could not increase the risk of developing testicular cancer, although an inverse relationship was observed in the group with the least exposure to PVC [91]. The results of these studies, summarized in Table 1, illustrate the conflicting evidence regarding the association between PVC exposure and testicular cancer.

**Table 1:** Studies on occupational PVC exposure and testicular cancer risk.

**Author, date**	**Type of Study**	**Population / Sample**	**Findings**	**Reference**
Hardell et al., 1997	Case-control	Workers exposed to PVC	PVC exposure increased risk of seminoma by 6X. Excluding orchitis/cryptorchidism cases further increased risk.	[18]
Langård et al., 2000	Cohort	Workers exposed to vinyl chloride	No increased risk of testicular cancer despite known PVC-related risks for other malignancies.	[89]
Hansen, 1999	Case-control	Plastics industry workers	No clear link between plastics exposure and testicular cancer. There may have been misclassification of groups in this study, potentially affecting the results.	[90]
Hardell et al., 2004	Case-control	Workers with varying levels of PVC exposure	No increased risk of testicular cancer with PVC exposure; an inverse relationship seen in low-exposure group.	[91]

## 6 The effect of microplastics on the spermatogenesis process

In humans, more abnormal sperms are produced than in some species of laboratory or domesticated animals, which shows the fundamental difference between the process of spermatogenesis in humans compared to other species. This condition may be due to the fact that the process of human spermatogenesis is more vulnerable to external factors. There is also the concern that sperm count has significantly decreased in the last half century. Of course, evidence also shows that sperm count may be subject to different environmental and ethnic-racial conditions [92], [93], [94]. The process of spermatogenesis does not begin until puberty, and after that, this process remains healthy throughout a man's life. Therefore, during this period of time, spermatogenesis can be impaired due to environmental factors as well as occupational exposure. Exposure to environmental chemicals (ECs) occurs in different ways, including the type of job, environmental conditions at home, and lifestyle. There is strong evidence that exposure to ECs can impair spermatogenesis and decrease sperm count in men [94]. Heavy metals can be mentioned among the pollutants that have a destructive effect on the process of spermatogenesis and sperm count [95]. Another group of environmental pollution is plastic materials, which entered human daily life since the 1950s. This type of pollution can also be absorbed by the body through different ways and enter the reproductive system of men and disrupt the process of spermatogenesis and sperm function [96]. Therefore, understanding the mechanisms of the effect of MPs on the process of spermatogenesis and ultimately male infertility can be very important.

### 6.1 The effect of microplastics on the sperm parameters and the integrity of the sperm genome

Polystyrene MPs can disrupt spermatogonia and reduce sperm quality, indicating potential harm to male reproductive function in animal models [52]. When pregnant mice are exposed to MPs, their offspring have damaged epithelium and reduced sperm [97]. Another study by Li et al. (2021) showed that PS-MPs can reduce the number and motility of sperm and increase abnormal sperm. It was found that the mentioned microplastic activates the p38 MAPK pathway and reduces the Nrf2 molecule and ultimately causes oxidative stress. Molecular pathways activated by polystyrene can induce apoptosis in spermatogenic cells [98]. In another study, it was found that PS-MPs can disrupt the morphology, motility, and number of sperms in mice. This microplastic can disrupt sperm metabolism by reducing the activity of lactate dehydrogenase and succinate dehydrogenase enzymes. They also found that polystyrene causes oxidative stress, which ultimately damages sperm [99]. The effect of PS-MPs on rat spermatogenesis showed that after exposure to this substance, the number of viable epididymis sperm was significantly reduced. Also, this exposure changed sperm morphology and increased sperm cell apoptosis. In this research, it was found that cellular signaling including pro-inflammatory molecules and inflammatory factors as well as the Nrf2/HO-1 pathway are involved [53].

Exposure to MPs can be related to sperm genome fragmentation. It is known that the fragmentation of sperm DNA causes sperm motility and morphology abnormalities (Fig. 3) [100]. Single-stranded and double-stranded DNA breaks may affect the fertilization process. Single-stranded and double-stranded DNA breaks may affect the fertilization process. It has been determined that if the seminal fluid samples of sperms with double-stranded and single-stranded DNA breaks are used for intracytoplasmic injection of sperm, it may affect the growth of the embryo and its implantation. A study by Casanovas et al. (2019) found that double-stranded DNA damage causes implantation failure and fetal growth retardation [101]. Sperm DNA integrity is determined by the DNA fragmentation index (DFI), which is much higher in infertile than in fertile men [102]. One study found a direct correlation between frequent pregnancy loss and fragmented sperm genome [103]. It has also been found that if the DFI level is high, it can cause a decrease in the fertility rate and an increase in abortion [104]. Therefore, sperm genome fragmentation is involved in the natural pregnancy process and can be used as a useful indicator to evaluate male reproduction in the clinic. Factors that cause sperm DNA fragmentation (SDF) are complex, and among these factors, old age or exposure to toxic substances can be mentioned [105], [106]. In a study, the effect of polystyrene on the infertility parameters of rats, including the integrity of their sperm genome, was investigated. The result of the study showed that exposure to certain doses of polystyrene can lead to DNA damage [107]. In another study, the effects of polystyrene on parameters affecting the fertilization success of Tegillarca granosa, including DNA integrity, were investigated. Their results showed that exposure to MPs causes sperm genome fragmentation. They suggested the possible mechanism of this phenomenon caused by the activation of some caspases induced by MPs. They concluded that this reduction in sperm genome integrity may reduce sperm motility and reduce sperm competition at fertilization [108].

### 6.2 The effect of microplastics on cells involved in spermatogenesis

MPs can disrupt the structure and function of germ cells, Leydig cells, and Sertoli cells, potentially impairing spermatogenesis [6], [109]. In a study, Hamza et al. (2023) investigated the harmful effects of polystyrene on reproductive indicators of mice, including germ cells. Apart from the fact that this microplastic caused structural disorder, exposure to polystyrene decreased the number of germ cells, spermatogonia, and primary spermatids in the exposure group compared to the control group. Of course, this destructive effect was significantly moderated by a natural substance called Rhamnetin. This substance induced a regular number of acceptable spermatogenic germ cells in mice [110]. Also, another study determined that the exposure of mice to polystyrene microplastic caused a significant decrease in the number of germ cells [111]. Exposure to MPs can cause oxidative stress, and this issue changes the expression of inflammatory factors and proteins and activates some cell signals, which in turn can cause a decrease in germ cells [112]. Therefore, disorders such as meiosis in spermatocytes and sperm maturation, which are necessary for the process of spermatogenesis, may be disturbed during exposure to MPs. So, considering the widespread use of MPs around the world and the great impact of these substances on men's reproductive health, it is very important to know the cellular mechanisms of disorders caused by MPs in germ cells, and extensive research in this field is necessary [113].

In addition to germ cells, Sertoli cells and Leydig cells also play a major role in the complex process of spermatogenesis. Sertoli cells, which are part of the BTB, are responsible for the nutritional and structural aspects of spermatogenesis. These cells can act as a protector to regulate the integrity of the germ cell microenvironment. Sertoli cells protect the germ cells against foreign toxic substances through cell junctions such as gap junctions and desmosomal junctions. It also regulates the growth of germ cells through these cell connections by relevant factors [114], [115]. Although the entry of xenobiotic substances is blocked through the BTB, however, small-sized substances are able to penetrate the seminiferous epithelium [116]. Factors such as MPs that cause damage to the BTB somehow act by disrupting the Sertoli cells. Therefore, in pathological conditions, the restrictions created by Sertoli cells are lost [117]. In a study conducted by Gao et al. (2023), the effect of exposure to MPs on the reproductive parameters of mice was investigated. Exposure to MPs causes reproductive toxicity and affects spermatocytes, spermatogonia and Sertoli cells. Exposure to MPs reduces the number of Sertoli cells. Ultrastructural analysis of Sertoli cells shows that MPs cause disturbances in cell junctions, interstitial edema and swelling of mitochondria [118]. Leydig cells, which produce androgens such as testosterone in the adult male testis and reveal secondary sexual characteristics, are susceptible to environmental pollution. Exposure to MPs in mice can reduce Leydig cell area, which was accompanied by a decrease in the number of cells and condensation of nuclei [118]. It should be noted that the effects of MPs on germ cells, Leydig cells, and Sertoli cells have primarily been investigated through in vitro experiments. These findings will be discussed in detail in the in vitro studies section.

## 7 The effect of microplastics on sex hormones and endocrine disorders

Many chemicals interfere with the function of hormones or their receptors as endocrine-disrupting chemicals (EDCs) and can cause endocrine and developmental disorders [119]. The analysis of human feces shows that there are 9 different types of MPs in it, which confirms the presence of MPs in their food chain [120]. MPs and sometimes substances with them can pass through the cell membranes and the BTB and interact with hormone receptors and subsequently disrupt the various hypothalamic axes, including the hypothalamic-pituitary-gonadal axis, the hypothalamic-pituitary-adrenal axis, and the hypothalamic-pituitary-thyroid axis [42], [121], [122], [123]. The wide and hydrophobic surface of MPs makes them suitable for absorbing many pollutants including heavy metals and EDCs and makes it possible for them to accumulate in different tissues [124]. Some additives include EDCs, which can be absorbed by MPs and enter the body and accumulate in different parts after entering the food chain directly or indirectly [125]. These particles interfere in different tissues and different cellular pathways and affect cellular functions depending on their size [126]. These particles can also enter the reproductive tissues and change the physiological function of the male reproductive system [127]. Considering the increasing spread of MPs in the environment and on the other hand the spread of reproductive disorders in men, studying and understanding the mechanisms of the effect of these particles on the male reproductive system has become more important. It is very important to expose animal models to MPs and investigate their harmful effects on the reproductive system and their effects on sex hormones, especially testosterone.

It has been found that long-term exposure of rats to PS-MPs can disrupt their reproductive system. A study by Jin et al. (2022) showed that PS-MPs can reduce serum levels of testosterone, LH, and FSH hormones. Polystyrene exerts its testosterone-lowering action through LH-mediated downregulation of the LHR/cAMP/PKA/StAR pathway [128]. In another study, exposure of mice to PS-MPs caused a decrease in serum testosterone content. Molecular analysis showed that the testosterone synthetic initiator, steroidogenic acute regulatory protein, has negative expression regulation. When endoplasmic reticulum stress (ERS) inhibitors were used, testicular damage was reduced and testosterone levels were also elevated. This study suggested that polystyrene may impair male reproduction by activating the apoptosis and ERS pathways [129]. In another study, the effects of PS-MPs on reproductive parameters of male rats were investigated. After short-term exposure of mice to polystyrene particles, these particles accumulated in the testicles of mice. Also, *in vitro* conditions, polystyrene in 3 different sizes was introduced into 3 types of testicular cells. Also, these particles caused reduced sperm quality and decreased testosterone levels [52]. Another group investigated the effects of polystyrene on the reproductive system of male rats, including testosterone levels. The results of their study also showed that the mentioned microplastic can reduce the level of testosterone [96]. In the study of Ijaz et al. (2021), the microplastic effect of polystyrene in different doses on the reproductive system of male rats was investigated. Their hormonal analysis showed that exposure to different doses of polystyrene can suppress the plasma level of LH. Also, at the highest dose of polystyrene, inhibition of plasma FSH was clearly observed. Plasma concentration of testosterone was similarly decreased at high concentration of MPs. The intratesticular concentration of testosterone was also inhibited by high doses of polystyrene [130]. In summary, exposure to MPs leads to alterations in male sex hormone levels, which are accompanied by measurable impairments in key reproductive parameters.

## 8 *In vitro* evidence of microplastics-induced testicular and sperm toxicity

*In vitro* studies have provided substantial evidence regarding the toxic effects of MPs on male reproductive cells and functions. Confocal microscopy demonstrated that TM4 Sertoli cells and GC-2 germ cells effectively internalized fluorescein-labeled PLA-MPs after 24 h of exposure. Transmission electron microscopy further revealed the penetration of PLA-MPs into the mitochondria of TM4 cells, accompanied by ultrastructural damage such as vacuolization and loss of mitochondrial cristae. CCK-8 assays confirmed increased cytotoxicity along with reduced cell proliferation and viability. Moreover, dose-dependent increases in mitochondrial calcium levels and ROS production, together with decreased mitochondrial membrane potential and ATP levels, were observed in both cell types, indicating mitochondrial dysfunction [61].

In another study, three testicular cell types, germ cells, Leydig cells, and Sertoli cells, were exposed for 24 h to fluorescent PS-MPs with particle sizes of 0.5, 4, and 10 µm. Immunofluorescence analysis showed that PS-MPs of all sizes were capable of penetrating and accumulating within testicular cells [52]. Similarly, *in vitro* exposure of spermatogonial cells to PS-MPs significantly increased apoptosis, reduced mitochondrial membrane potential, disrupted mitochondrial structure, and elevated mitochondrial ROS generation, collectively indicating the induction of severe oxidative stress [58].

A mechanistic study investigated the effects of PS-MPs on cultured TM3 Leydig and TM4 Sertoli cells, focusing on mitochondrial function and endoplasmic reticulum (ER) crosstalk. PS-MP exposure significantly reduced cell viability in both cell lines. In TM3 cells, PS-MPs suppressed the expression of steroidogenic acute regulatory protein and key steroidogenic enzymes, including 3β-HSD and 17β-HSD. In TM4 cells, reduced androgen receptor expression and lactate dehydrogenase activity were observed. Additionally, PS-MPs impaired ERK1/2 and Akt phosphorylation, induced oxidative stress, activated autophagy and apoptosis, and caused mitochondrial dysfunction and ER stress. These findings indicate that PS-MPs disrupt the functional integrity of testicular somatic cells, thereby contributing to male infertility through impaired steroidogenesis and compromised Sertoli cell support functions [131].

Other *in vitro* study using mouse TM4 Sertoli cells demonstrated that PS-MPs penetrated the cellular lumen within 1 h of exposure, with intracellular accumulation increasing over 24, 48, and 72 h. Western blot analysis revealed significant upregulation of inflammatory mediators, including IL-6, TNF-α, MCP-1, TGF-β, IL-10, and HIF-2α, whereas no significant increase in oxidative stress markers was detected [132]. In a study, TM3 cells were exposed to polystyrene microplastic *in vitro*. The results of the study showed that MPs can enter cells and cause cytotoxicity by causing mitochondrial dysfunction and oxidative stress. These can interfere with cellular processes such as apoptosis, testosterone biosynthesis, mitochondrial genome replication and energy metabolism. Also, polystyrene causes destruction and leakage of cell membrane [133].

Another study using human semen samples, exposure to PS-MPs resulted in time- and dose-dependent reductions in sperm motility and viability, while sperm concentration and morphology remained unchanged. Increased sperm agglutination, elevated ROS production, and higher SDF were also observed. Furthermore, decreased expression of genes involved in sperm-oocyte fusion suggested that PS-MP exposure may impair the fertilization capacity of human spermatozoa [134]. To provide an overview, a summary of the results of the in vitro studies discussed is presented in Table 2.

**Table 2:** In vitro evidence of microplastic effects on male reproductive cell types.

**Author, date**	**Type of microplastic**	**Cell type**	**Findings**	**Reference**
Zhao et al., 2025	Polylactic acid (PLA)	TM4 Sertoli and GC-2 germ cells	PLA microplastics were internalized by TM4 Sertoli and GC-2 germ cells, causing mitochondrial damage, increased cytotoxicity and ROS production, and reduced ATP and mitochondrial membrane potential.	[61]
Fang et al., 2024	Polystyren (PS)	GC-1 mouse spermatogonium	Exposure of spermatogonial cells to PS microplastics significantly increased apoptosis, disrupted mitochondrial structure, reduced mitochondrial membrane potential, and elevated ROS production.	[58]
Jin et al., 2020	Polystyren	Germ, Leydig, and Sertoli cells	PS microplastics (0.5, 4, and 10 µm) penetrated and accumulated in germ, Leydig, and Sertoli cells after 24 h of exposure.	[52]
Grillo et al., 2024	Polystyren	TM3 Leydig and TM4 Sertoli cells	Microplastics reduced cell viability in TM3 Leydig and TM4 Sertoli cells, suppressing steroidogenic enzymes, androgen receptor expression, and lactate dehydrogenase activity. َAlso, they induced oxidative stress, autophagy, apoptosis, mitochondrial and ER stress.	[131]
Jeon et al., 2024	Polystyren	TM4 Sertoli cells	PS microplastics rapidly entered TM4 Sertoli cells and accumulated intracellularly over time. Exposure significantly increased inflammatory mediators without inducing oxidative stress.	[132]
Sun et al., 2023	Polystyrene	TM3 Leydig cells	Microplastics can enter cells and cause cytotoxicity by causing mitochondrial dysfunction and oxidative stress. Also, polystyrene causes destruction and leakage of cell membrane.	[133]
Mottola et al., 2025	Polystyrene	Sperm	Exposure to PS caused dose- and time-dependent reductions in sperm motility and viability, while sperm concentration and morphology remained unchanged. Increased ROS levels, sperm agglutination, and DNA damage indicated impaired fertilization capacity.	[134]

## 9 Solving the challenges of dealing with microplastics

Considering the many problems that MPs have created for the environment and human societies, scientists are trying to discover or improve ways to eliminate plastic pollution. Nowadays, the removal of MPs is not easy, but it can be done using modern physical, chemical and biological methods. In general, changing behavioral habits and limiting the use of plastic to prevent the increase in the production of microplastic waste is an approach that should be widely adopted. Using biodegradable plastics in food packaging, using cotton and linen clothes instead of polyester, nylon and polystyrene, limiting the use of single-use containers and banning the use of MPs in cosmetics and detergents are among the actions that society members and governments are encouraged to adopt in order to protect the environment [8], [135].

Among the ways to remove microplastic pollution from the environment is the use of physical methods such as rapid sand filtration, adsorption, photocatalytic decomposition, electrocoagulation and magnetic absorption [8]. The rapid sand filtration method is a powerful process to remove pollutants from the wastewater and it is possible to remove MPs with this method to a large extent, but even so, some MPs may pass through the sand filters. To increase the efficiency of this method, electrical, chemical or electrochemical coagulation methods are used [136], [137]. Another approach for MP removal is the adsorption process, in which physicochemical interactions enable materials such as chitin, biochar, graphene oxide, and chemically synthesized sponges to capture MPs and nanoplastics. Sponges with their active side chains can absorb MPs to a great extent. However, by removing absorbent materials, the possibility of pollutants returning to the environment is very high. In order to reduce the risk of pollutant return, graphene oxide and chitins are used to make the pollutant binding to the adsorbents more stable. In addition, magnetic biochar materials can promote the removal and partial thermal degradation of MPs through heat treatment, thereby reducing the risk of secondary environmental contamination [138]. In the photocatalytic process, ultraviolet or visible light is absorbed by semiconductor materials, leading to the generation of reactive radicals that induce surface oxidation and partial degradation of MPs [139].

Another option to remove MPs is to use chemical methods. These methods include charge neutralization, surface absorption, and flocculation, which is most used is related to the flocculation method and is considered as one of the most important approaches to urban wastewater treatment. Various coagulants, including aluminum and iron-based materials, are used in this method [140]. Even though the coagulation method is one of the most common water treatment approaches, it comes with major challenges. The use of different coagulants due to the simultaneous presence of different pollutants in water and the need for additives to increase the efficiency of coagulants are among the factors that increase the overall cost of this method. One of the most important problems of using this method is that a large amount of sludge is produced as a result of the coagulation and precipitation of pollutants. Sludge production is very problematic because the sediments have a high density and may contain much more dangerous pollutants than the original pollution. This problem has caused the release of these pollutants into the environment and has increased the cost of removing secondary pollution [8]. Today, to overcome some of these challenges, there are solutions, for example, they use coagulants that produce less sludge. Among these coagulants, we can mention polyaluminum chloride, which produces less sludge than AlCl_3_, which was used as a coagulant in the past. Another reason for the superiority of this substance is its lower dependence on temperature and pH, as well as its ability to be used in lower doses [141].

Among other chemical methods of removing MPs is electrochemical oxidation, which is done in two ways: anodic oxidation and indirect cathodic oxidation, where anodic oxidation is more common. In this method, pollutants are absorbed directly, through charge transfer, to the surface of the anode, or indirectly by charged elements such as chlorine to the surface of the cathode [142]. In this method, various pollutants such as MPs, antipyretics, antibiotics, and dyes, undergo oxidative degradation and transformation into less toxic byproducts, with partial mineralization occurring under optimized conditions. In addition, another advantage of using this method is that the free radicals produced during the process prevent the creation of sludge [8]. The efficiency of this method depends on various factors, including the contact surface, the materials used on the anode surface, the type and concentration of the electrolyte used, and the duration of the decomposition reaction. In summary, this is the approach that has garnered the interest of researchers, and their objective is to enhance the methodologies employed in implementing this technique [143].

Using biological methods to remove MPs is a relatively recent approach that has attracted the attention of many scientists today. Using membrane bioreactor technology is one of the methods of biological removal of MPs. In this method, different microorganisms are used, including nitrifying bacteria, and this method is one of the most efficient methods of industrial and urban wastewater treatment [144]. In this method, a combination of biological processes and the use of membrane filters are used in a chamber. In this process, pollutants and particles suspended in water are separated by membranes with nanometer pores, and with the presence of microorganisms in the solution inside the tank, these pollutants are biologically treated or partially degraded [145]. This method has many characteristics such as the high power of removing the target pollutants and the high efficiency of pollutant removal as well as the high quality of the effluent. Among the disadvantages of this method are the need for continuous feeding of the microorganisms used, the blocking of the membrane pores with sediments, and the limitation of space, which lead to the expensiveness of this method [8], [145]. Other biological approaches include the use of algae and fungi that induce partial biodegradation or surface modification of MPs, as well as genetically modified microorganisms, all of which are still under investigation [135].

## 10 Conclusion and future perspectives

Nowadays, microplastics are recognized as an emerging environmental concern with significant implications for human health. These particles enter the human body through ingestion, inhalation, and to some extent dermal contact, and can accumulate in various tissues. Evidence suggests that the presence of MPs in the body is associated with a range of disorders, including cancer, neurological abnormalities, and reproductive problems. In the male reproductive system, these particles are capable of penetrating testicular tissue and affecting its structure and function. As a consequence, disruptions in spermatogenesis, reductions in sperm quality, and ultimately an increased risk of male infertility may occur. At the cellular and molecular levels, MPs contribute to these adverse effects by inducing oxidative stress, triggering inflammatory responses, activating apoptotic pathways, and disrupting the balance of critical cellular signaling pathways, thereby playing a significant role in functional impairment of reproductive tissues.

Despite the growing body of evidence highlighting the adverse effects of MPs on male reproductive health, several critical knowledge gaps remain, underscoring the need for further research. Advanced analytical techniques are required to better characterize MPs within biological systems. The development of sensitive and standardized methods for the detection, quantification, and characterization of MPs in human tissues, biological fluids, and reproductive organs remains a major challenge. Another important future direction involves elucidating the molecular and epigenetic mechanisms underlying MP-induced reproductive toxicity. Although oxidative stress, mitochondrial dysfunction, inflammation, and endocrine disruption have been implicated, their interactions with epigenetic alterations, such as DNA methylation, histone modifications, and non-coding RNA regulation, remain largely unexplored. These mechanisms may contribute to intergenerational reproductive effects and long-term fertility impairment.

Moreover, human-based studies, including epidemiological investigations and ex vivo analyses of human reproductive tissues and semen samples, are urgently needed. Such studies would help establish causal relationships between MP exposure and male infertility, reduced semen quality, and hormonal imbalances in real-world populations. Finally, future research should move beyond mere toxicity assessment and simultaneously focus on exposure mitigation strategies and post-exposure interventions. Exposure reduction strategies include the development of safer alternatives to conventional plastics and improvements in waste management policies, whereas post-exposure interventions may involve exploring antioxidant, anti-inflammatory, and lifestyle-modification approaches to alleviate microplastic-induced damage. Integrating toxicological data with environmental science, public health, and regulatory frameworks is essential to effectively address the global challenge of MPs and to safeguard the reproductive health of future generations.

## Abbreviations

BTB, Blood-testis barrier; DFI, DNA fragmentation index; ECs, Environmental chemicals; EDCs, Endocrine-disrupting chemicals; ER, Endoplasmic reticulum; ERS, Endoplasmic reticulum stress; MPs, Microplastics; PE-MP, Polyethylene microplastics; PET, Polyethylene terephthalate; PLA, Polylactic acid; PS, Polystyren; PS-MPs, Polystyrene microplastics; PVC, Polyvinyl chloride; ROS, Reactive oxygen species; SDF, Sperm DNA fragmentation.

## CRediT authorship contribution statement

**Mohammad Karimian:** Writing – review & editing, Writing – original draft, Supervision, Methodology, Investigation. **Sahar Yaqubi:** Writing – review & editing, Writing – original draft, Investigation.

## Consent to participate

N/A.

## Consent for publication

This manuscript has been submitted with the full agreement and approval of all contributing authors.

## Ethical approval

The authors confirm that this article does not involve any research conducted on human or animal subjects.

## Declaration of Generative AI and AI-assisted technologies in the writing process

AI tools were used only for language editing and grammar checking.

## Funding

None.

## Declaration of Competing Interest

The authors declare that they have no known competing financial interests or personal relationships that could have appeared to influence the work reported in this paper.

## Data Availability

No data was used for the research described in the article.
